# Characterization and Antihyperglycemic Activity of a Polysaccharide from *Dioscorea opposita* Thunb Roots

**DOI:** 10.3390/ijms16036391

**Published:** 2015-03-19

**Authors:** Yijun Fan, Qinyi He, Aoshuang Luo, Miaoyu Wang, Aoxue Luo

**Affiliations:** 1 Department of Landscape Plants, Sichuan Agriculture University, Chengdu 611130, China; E-Mails: qinyihe@yeah.net (Q.H.); miaoyuwang0@126.com (M.W.); 2 Chengdu Institute of Biology, Chinese Academy of Sciences, Chengdu 610041, China; E-Mail: luoshuang100@163.com

**Keywords:** *Dioscorea opposita* Thunb, antihyperglycemic, polysaccharide

## Abstract

A polysaccharide DOTP-80 from *Dioscorea opposita* Thunb was obtained by using the method of acid water-extraction and ethanol-precipitation. After being purified by chromatography, the structure characteristics of DOTP-80 were established. Based on the calibration curve obtained with standard dextrans, the molecular weight of the polysaccharide fraction DOTP-80 was calculated to be 123 kDa. The results of Infrared spectrum (FT-IR) indicated that the polysaccharide contained the α-configuration of sugar units. GC-MS analysis revealed that DOTP-80 was mainly composed of mannose and glucose. Alloxan-induced diabetic rats and mice models were developed to evaluate the *in vivo* hypoglycemic activity of the polysaccharide. The results indicated that a high dose DOTP-80 (400 mg/kg) had strong hypoglycemic activity. Moreover, DOTP-80 could increase the level of antioxidant enzymes (SOD) activity in alloxan-induced diabetic mice and stimulate an increase in glucose disposal in diabetic rats. Therefore, the polysaccharide DOTP-80 should be evaluated as a candidate for future studies on diabetes mellitus.

## 1. Introduction

Diabetes is a chronic metabolic disease characterized by hyperglycemia, which results from an absolute or relative deficiency of insulin secretion [1]. Though various types of oral hypoglycemic agents are available along with insulin for the treatment of diabetes mellitus so far, many drugs are limited by side effects and toxicity [2]. Therefore, it is important to investigate the new nontoxic hypoglycemic drugs. As the compounds from plants, originally applied in traditional medicine, are usually relatively nontoxic and do not produce significant side effects [3,4], research into hypoglycemic drugs in plants is a hot topic. Numerous plants possess abundant polysaccharide compounds; many of these polysaccharides play key biological roles in life processes [5], which were often associated with immunomodulatory effects [6,7], antitumor effects [8,9], antioxidant activity [10,11], anti-inflammatory activity [12,13], anti-virus activity [14], and especially hypoglycemic activity [15,16,17]. *Dioscorea opposita* Thunb is an edible and traditional medicinal plant in China [18] and the tuber is often used to treat poor appetite, chronic diarrhoea, asthma, dry coughs, frequent or uncontrollable urination and diabetes [19]. There are abundant polysaccharides in *Dioscorea opposita* Thunb [20]. But, until now, the purification and hypoglycemic activity of the polysaccharide from *Dioscorea opposita* Thunb has rarely been reported. Therefore, the purpose of the present work is to elucidate the isolation and characterization of water-soluble polysaccharide from *Dioscorea opposita* Thunb, as well as to evaluate its hypoglycemic activity.

## 2. Results and Discussion

### 2.1. Polysaccharide Isolation and Purification

The crude polysaccharide DOTP-80 was obtained by means of water-extraction and ethanol-precipitation. As there were some colored materials and residual protein, the crude polysaccharide was yellow water-soluble powder. AB-8 and ADS-7 macroporous resins were employed to further purify the polysaccharide. After the application of these two macroporous adsorption resins, the polysaccharide presented off white-colored powder.

The molecular weight of polysaccharide is an important factor of biological activities, therefore, determining the molecular weight is the first step of the study. Based on the calibration curve obtained with standard dextrans, the molecular weight (*M*_W_) of the polysaccharide fraction DOTP-80 was calculated to be 123 kDa. Trifluoroacetic acid hydrolysis and GC-MS analysis method were adopted to analyze the monosaccharide compositions of the polysaccharide fraction. The results indicated that glucose and mannose were major monosaccharides forming the backbone of DOTP-80. The molar ratio of monosaccharide compositions in DOTP-80 was described as follows: glucose:galactose:mannose:arabinose = 23.7:9.3:17.8:1.0.

### 2.2. Infrared Spectrum of the Polysaccharide

**Figure 1:**
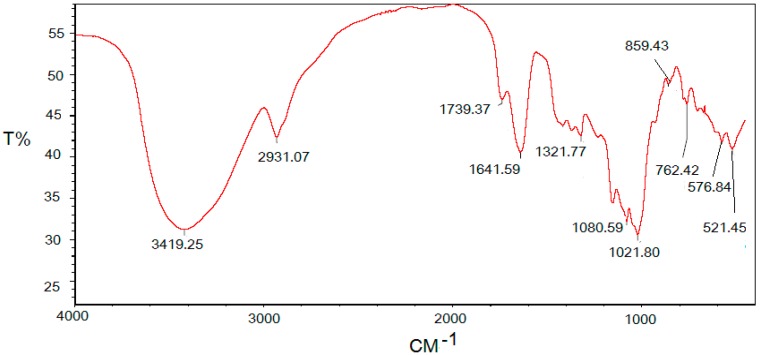
IR spectrum of the polysaccharide DOTP-80.

The FT-IR spectrum of the polysaccharide DOTP-80 was presented in Figure 1; the band at 3419.2 corresponds to the hydroxyl stretching vibration and the band at 2931.0 corresponds to a weak C–H stretching vibration [21], which indicated that the sample was a polysaccharide compound. The band at 1641.5 was due to the bound water [22]. In addition, the polysaccharide had a specific band in the 1200–1000 cm^−1^ region and this region was dominated by ring vibrations overlapped with stretching vibrations of (C–OH) side groups and the (C–O–C) glycosidic band vibration [23]. Positive specific rotation and the characteristic absorption at 859.4 cm^−1^ in indicated the α-configuration of the sugar units [24].

### 2.3. Effect of Polysaccharide on the Blood Glucose Level in Healthy Mice

The effect of DOTP-80 on plasma glucose level in healthy mice was shown in Table 1. Before administration, there was no significant difference in blood glucose levels among all groups. Furthermore, no significant difference was observed between the basal glucose levels in both the control (isotonic saline solution) and polysaccharides groups. However, a significant reduction of plasma glucose level was observed after the administration of Glipizide. The results indicated that the polysaccharide DOPT-80 could not stimulate the secretion of insulin, and also suggested treatment of diabetics with the polysaccharide could not cause hypoglycemia.

**Table 1:** Effects of DOTP-80 on plasma glucose level of normal mice.

Groups	Dose (mg/kg Body wt.)	Plasma Glucose Level (mmol/L)			
		Day 0	Day 6	Day 12	Day 18
Control	-	5.3 ± 0.9	5.5 ± 0.6	5.3 ± 0.7	5.5 ± 0.7
Glipizide	200	5.5 ± 0.4	5.1 ± 0.8 *	4.8 ± 1.0 *	4.1 ± 0.3 **
DOTP-80	400	5.3 ± 0.6	5.3 ± 1.0	5.4 ± 0.5	5.6 ± 0.7
	200	5.5 ± 0.7	5.5 ± 0.6	5.5 ± 0.9	5.4 ± 0.8
	100	5.5 ± 0.9	5.3 ± 0.7	5.2 ± 0.5	5. 6 ± 0.4

Each value represents mean ± SD (*n* = 10); * *p* < 0.05 and ** *p* < 0.01 (compared with control).

### 2.4. Effect of Polysaccharide on the Blood Glucose Level in Alloxan-Induced Diabetic Mice

The effect of DOTP-80 on plasma glucose level in alloxan-induced diabetic mice was shown in Table 2. Before administration, there was no significant difference in blood glucose levels among all groups. After six days, there was a significant (*p* < 0.05) decrease in the metformin hydrochloride group when compared with the model control group. Furthermore, when compared with the diabetic control group, the daily administration of DOTP-80 (200 and 400 mg/kg) for 12 and 18 days in alloxan-induced diabetic mice led to a significant decline in the blood glucose level (*p* < 0.05). Nevertheless, the hypoglycemic activity in the low dose group (100 mg/kg DOTP-80) was weak. According to the table, all samples exhibited obvious effects in a concentration-dependent manner. Therefore, these results clearly revealed that high dose DOTP-80 possessed potential hypoglycemic activity in alloxan-induced diabetic mice.

**Table 2:** Effect of DOTP-80 on plasma glucose level in alloxan-induced diabetic mice.

Groups	Dose (mg/kg Body wt.)	Plasma Glucose Level (mmol/L)			
		Day 0	Day 6	Day 12	Day 18
Control	-	20.9 ± 3.1	21.8 ± 1.9	21.0 ± 2.8	22.1 ± 3.3
Metformin hydrochloride	200	21.3 ± 2.5	17.6 ± 1.3	13.8 ± 1.7 **	10.3 ± 2.0 **
DOTP-80	400	20.1 ± 2.6	18.7 ± 2.4	15.4 ± 0.5 *	12.1 ± 1.9 **
	200	20.1 ± 2.0	19.6 ± 1.9	16.7 ± 2.5 *	13.3 ± 2.2 **
	100	21.1 ± 2.4	20.0 ± 2.4	18.4 ± 2.7	15.5 ± 2.7 *

Each value represents mean ± SD (*n* = 10); * *p* < 0.05 and ** *p* < 0.01 (compared with model control).

### 2.5. Effect of DOTP-80 on Body Weight in Mice

**Figure 2:**
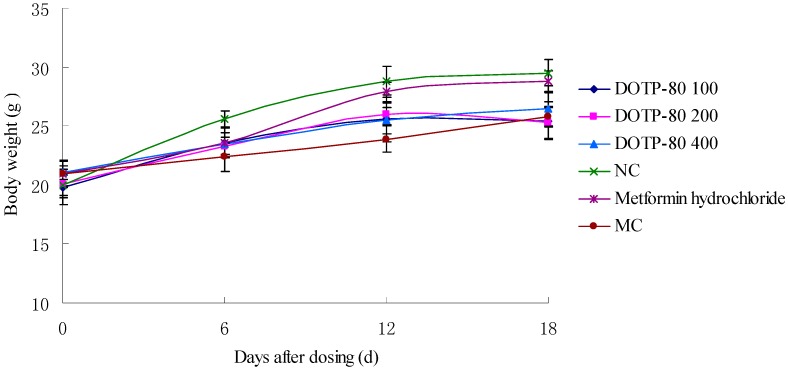
Effect of different doses (mg/kg body weight) of DOTP-80 and Metformin hydrochloride on body weight in alloxan-induced diabetic mice. NC: normal control group; MC: model control.

The effect of DOTP-80 on body weight was assessed in alloxan-induced diabetic mice (Figure 2). Before initiating on the experiments, the body weights in all the groups were 20.0 ± 2.0 g. After the intragastric drug administration, a significant (*p* < 0.05) decrease in body weight was detected in all the diabetic groups when compared with the normal control (NC) group 12 days later. Moreover, six days after administration, the body weight in the DOTP-80 group significantly (*p <* 0.05) and dose-dependently increased when compared with that in the MC group. The results showed that there was no significant toxic effect of the polysaccharide DOTP-80 on the mice.

### 2.6. Effect of the Polysaccharide on Blood Glucose Levels in Alloxan-Induced Diabetic Rats

According to the results above, high dose of DOTP-80 (400 mg/kg) exhibited the strongest hypoglycemic activity among the three concentrations. Therefore, oral glucose tolerance test (OGTT) assays were performed conducted in the alloxan-induced diabetic rats to investigate the hypoglycemic activity of the polysaccharide DOTP-80 (400 mg/kg) in depth. The effect of DOTP-80 on blood glucose level in rats is shown in Table 3. There was no significant difference in blood glucose levels among all groups before administration. However, 0.5 h after sugar administration, the plasma glucose reached the highest level in all the groups and then began to diminish. Two hours after sugar administration, the plasma glucose concentrations were back to normal levels or lower than those before administration. At 0.5 and 2 h, the blood glucose values in the metformin hydrochloride group were far lower than those in the normal group. Meanwhile, DOTP-80 exhibited a strong hypoglycemic effect. The blood glucose values at 2 h were close to those in the positive control (*p* < 0.05). Therefore, the results were an indication of DOTP-80 stimulating an increase in glucose disposal, which can be interpreted as a significant effect for stimulating the secretion of insulin in DOTP-80 at high concentrations.

**Table 3:** Effect of DOTP-80 on glucose tolerance of alloxan-induced rats.

Groups	Dose (mg/kg Body wt.)	Plasma Glucose Level (mmol/L)		
		0 h	0.5 h	2 h
Control	-	12.6 ± 1.8	25.6 ± 4.2	15.0 ± 4.0
Metformin hydrochloride	200	11.8 ± 2.2	13.2 ± 2.8 **	7.9 ± 1.4 **
DOTP-80	400	12.4 ± 2.6	15.6 ± 2.9 **	9.9 ± 2.3 **

Each value represents mean ± SD (*n* = 10); ** *p* < 0.01 (compared with model control).

### 2.7. Effect of the Polysaccharide on SOD Activity in Alloxan-Induced Diabetic Mice

**Figure 3:**
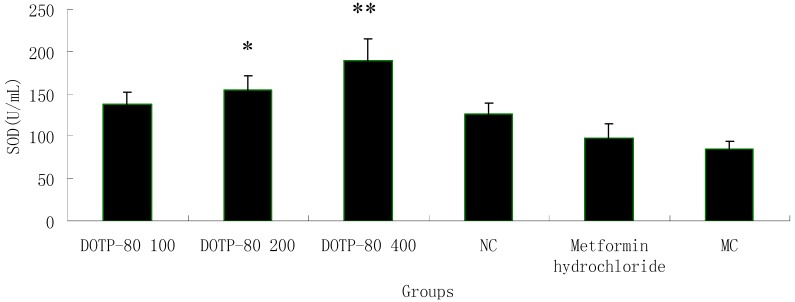
SOD activity. Results are presented as means ± standard deviations. * *p* < 0.05 and ** *p* < 0.01 (compared with normal control).

Diabetes is a chronic metabolic disease associated with oxidative damage. Therefore, prevention of oxidative damage through natural antioxidants and control of postprandial hyperglycemia are important diabetic prevention strategies. In order to investigate the antioxidant activity of the polysaccharide DOTP-80, the SOD activity in alloxan-induced diabetic mice was tested. The results are shown in Figure 3. SOD activities of different doses of DOTP-80 exhibited dose-dependent behaviors. At 400 mg/kg, DOTP-80 exhibited a high SOD activity of 189.8 U/mL, which was far higher than that of the normal control (125.97 U/mL) (*p* < 0.05). However, the SOD activity at low concentrations (100 mg/mL) was less evident, which was similar to that of the model control. Hence, the results were an indication of enhancing SOD activity of DOTP-80 for high concentrations.

## 3. Experimental Section

### 3.1. Materials and Chemicals

Alloxan was purchased from Sigma (St. Louis, MO, USA). Dextrans of different molecular weights were purchased from Pharmacia Co. (Uppsala, Sweden). The standard monosaccharides (glucose, mannose, rhamnose, galactose, xylose and arabinose) were purchased from Chinese Institute for the Control of Pharmaceutical and Biological Products (Beijing, China). SOD Assay was purchased from Institute of Biological Engineering of Nanjing Jianchen (Nanjing, China), AB-8 and ADS-7 were purchased from the Chemical Plant of Nankai University (Tianjin, China). Glipizide was purchased from Hainan Jinxiao (Harbin, China). Metformin hydrochloride was purchased from Beijing Jiade. Trifluoroacetic acid (TFA), pyridine, methanol, acetic acid, ethanol, acetic anhydride and all other chemicals and reagents were analytical grade.

### 3.2. Extraction of Crude Polysaccharide from Dioscorea opposita Thunb

The method of Luo *et al.* [25] was applied to extract the crude polysaccharide. First, the *Dioscorea opposita* Thunb was thoroughly washed with water, and then pulverized by sonifier cell disrupter. Afterwards, the *Dioscorea opposita* Thunb was extracted in six times the volume (*v*/*m*) of 0.4% HCl for 5 h at room temperature and filtered with gauze. The residue was further extracted with 0.4% HCl three times. After being combined and concentrated, the above extracts were neutralized with NaOH and filtered. The filtrates were collected and deproteinized four times using the Sevag reagent [26]. The above extract was precipitated by adding ethanol (1.5 times the volume of aqueous extract) at 4 °C for 24 h, followed by centrifugation at 4000 rpm for 20 min. Then the solution was successively precipitated by adding ethanol until the concentration of ethanol reached 80%, which yielded the polysaccharide DOTP-80. After being washed successively with ethyl acetate and acetone, the polysaccharide was dissolved in deionized water and subsequently dialyzed against deionized water for 72 h and lyophilized.

### 3.3. Purification of the Crude Polysaccharide

Purification of the crude polysaccharide was made according to the method of Luo *et al.* [25], with some modifications. First, the polysaccharide DOTP-80 was dissolved in dd H_2_O. After centrifugation and membrane filtration with 0.45 μm Nucleopore filter, the supernatant was chromatographed in a column (26 mm × 300 mm) of AB-8 for the decoloration of the crude polysaccharide. Sequentially, the fraction was chromatographed in column (26 mm × 300 mm) of ADS-7 to remove the residuary protein. The columns were eluted with distilled water. Protein content was determined according to the method of Bradford *et al.* [27], with bovine serum albumin as standard.

### 3.4. Determination of the Molecular Weight of Purified Polysaccharide

The molecular weight of the polysaccharide was determined by Gel Permeation Chromatography according to the method of Yamamoto *et al.* [28], in combination with a Waters HPLC (Waters 515, Milford, MA, USA) equipped with an Ultrahydrogel Linear Column (300 mm × 7.8 mm). The column was eluted with 0.2 M phosphate buffer (pH 7.0) at a flow rate of 0.7 mL/min and detected by a Waters 2410 refractive index detector (RID). Dextran standards with different molecular weights (2500, 4600, 7100, 10,000, 21,400, 41,100, 84,400, 133,800, 200,000 Da) were used to plot the calibration curve.

### 3.5. Infrared Spectrum Analysis

FTIR spectrum of the polysaccharide DOTP-80 was collected with a Fourier trans-formed infrared spectrometer (Perkin-Elmer Corp., Waltham, MA, USA) in the wave range of 4000–500 cm^−1^ using the KBr-disk method [29].

### 3.6. Analysis of Monosaccharide Composition

Monosaccharide composition of the polysaccharide DOTP-80 was determined by GC-MS (QP2010, Shimadzu, Kyoto, Japan). Firstly, 10.0 mg polysaccharide was hydrolyzed with 2.0 M TFA at 110 °C for 4 h in a sealed glass tube. The hydrolyzed product was then prepared for acetylation. The acetylation was carried out with 10 mg of hydroxylamine hydrochloride and with 0.5 mL of pyridine by heating in a water bath for 30 min at 90 °C. After incubation, the mixture was cooled at room temperature, and then 0.5 mL of acetic anhydride was added and mixed thoroughly through vortexing. The tube was sealed and incubated in a water bath for another 30 min at 90 °C. After cooling, approximately 1 μL of clear supernatant was loaded onto an Rtx-5SilMS column (30 m × 0.32 mm) of the GC-MS. Alditol acetates of authentic standards (glucose, mannose, rhamnose, galactose, xylose, and arabinose) with myo-inositol (2 mg) as the internal standard were prepared and subjected to GC-MS analysis separately in the same way [30].

### 3.7. Hypoglycemic Activity Test in Vivo

#### 3.7.1. Effect of Polysaccharide on Fasting Blood Glucose Levels in Mice

Kunming mice approximately 18 to 22 g, were housed under normal laboratory conditions, *i.e.*, room temperature, 12/12 h light-dark cycle with free access to standard rodent chow and water. The study was carried out according to the “Principles of Laboratory Animal Care” (World Health Organization (WHO) Chronicle, 1985). After 18 h of fasting, the mice were injected with a freshly prepared aqueous solution of 2% alloxan monohydrate (200 mg/kg body weight) [31]. Orbital blood was taken out to determine fasting blood glucose 72 h later. The mice with blood glucose value above 11.1 mmol/L were regarded as diabetic mice model. All animals were treated daily for 18 days. The mice were treated as following: positive control received Glipizide (200 mg/kg body weight) for healthy mice and metformin hydrochloride (200 mg/kg body weight) for alloxan-induced diabetic mice, model control group (normal saline) and the polysaccharides (100, 200 and 400 mg/kg body weight). All the groups received drug administration daily by intragastric (i.g.).

Blood glucose levels were determined after 12 h of fasting, 2 h after the last administration of drugs. Blood samples were taken immediately by excising the eyeballs of the sacrificed animals, and serum insulin levels were determined. At the same time, the body weight of each mouse was measured by balance.

#### 3.7.2. Effect of the Polysaccharide on Blood Glucose Levels in Alloxan-Induced Diabetic Rats (OGTT)

SD rats, 220–250 g, were divided into three groups and there were ten animals in each group, which were injected with a freshly prepared aqueous solution of 2% alloxan monohydrate (200 mg/kg body weight). Orbital blood was taken out to determine fasting blood glucose 72 h later. The mice with blood glucose value above 11.1 mmol/L were regarded as diabetic mice model. All animals received oral dosing daily for 18 days. Group 1 received physiological saline as a control, group 2 received metformin hydrochloride (200 mg/kg body weight.) as a positive control; and group 3 received 400 mg/kg body weight of polysaccharide DOTP-80. Blood samples were taken immediately by excising the eyeballs of the sacrificed animals before the first administration and 0.5 and 2.0 h after the last administration of drugs. Glucose levels were determined by the method described above.

#### 3.7.3. Effect of the Polysaccharide on SOD Activity in Alloxan-Induced Diabetic Mice

Antioxidant enzymes are considered to be a primary defense for preventing biological macromolecules from being damaged. SOD is an important enzymatic antioxidant enzyme in antioxidant systems. In order to test the effect of the polysaccharide DOTP-80 on antioxidant enzyme, the SOD activities of serum in alloxan-induced diabetic mice were measured by using the SOD assay kit.

### 3.8. Statistical Analysis

The data were presented as mean ± standard deviation. Statistical analysis was conducted with the SPSS 16.0 software package (Chicago, IL, USA).

## 4. Conclusions

In our current study, a polysaccharide DOTP-80 from *Dioscorea opposita* Thunb was obtained by using the methods of acid water-extraction and ethanol-precipitation. The molecular weight of DOTP-80 was 123 kDa based on the calibration curve obtained with standard dextrans. The results of FT-IR indicated that the polysaccharide contained the α-configuration of sugar units. GC-MS analysis revealed that the molar ratio of monosaccharide compositions of DOTP-80 was glucose:galactose:mannose:arabinose =23.7:9.3:17.8:1.0. The results above also indicated high dose DOTP-80 (400 mg/kg) possessed potential hypoglycemic activity in alloxan-induced diabetic mice. Moreover, DOTP-80 could not stimulate an increase in glucose disposal in alloxan-induced diabetic rats instead of the secretion of insulin in healthy mice, but could stimulate an increase in glucose disposal in alloxan-induced diabetic rats. Therefore, the polysaccharide DOTP-80 should be considered as a candidate for future studies on diabetes. In addition, the polysaccharide DOTP-80 was found to increase the levels of antioxidant enzymes (SOD) activity in alloxan-induced diabetic mice. It was considered to play an important role as a dietary radical scavenger for the prevention of oxidative damage of pancreatic β-cell. In order to obtain more information, further investigation on the cellular mechanism of hypoglycemic activity will be carried out.
